# Supplementary material for: Impact of the COVID-19 Pandemic on Elderly Patients with Spinal Disorders
Source: J Clin Med. 2022 Jan 25;11(3):602. doi: 10.3390/jcm11030602 (PMC8836518; doi:10.3390/jcm11030602)
Supplement: Supplementary file 1 [file jcm-11-00602-s001.zip › jcm-1516563-supplementary.pdf]

Questionnaire items

Age

Sex

Did you hesitate to visit the hospital due to the COVID-19 pandemic?

- Hesitated
- Did not hesitate

Has your symptom improved as a result of self-quarantine due to the COVID-19 pandemic?

- Improved
- Stable
- Deteriorated
- Not answered

How has your exercise habit changed as a result of the COVID-19 pandemic?

- Increased
- Stable
- Decreased
- No exercise habit

EQ-5D-5L: Under each heading, please tick the ONE box that best describes your health before pandemic and TODAY.

- MOBILITY
  - I have no problems in walking about
  - I have slight problems in walking about
  - I have moderate problems in walking about
  - I have severe problems in walking about
  - I am unable to walk about
- SELF-CARE
  - I have no problems washing or dressing myself
  - I have slight problems washing or dressing myself
  - I have moderate problems washing or dressing myself
  - I have severe problems washing or dressing myself
  - I am unable to wash or dress myself
- USUAL ACTIVITIES (e.g. work, study, housework, family or leisure activities)
  - I have no problems doing my usual activities
  - I have slight problems doing my usual activities
  - I have moderate problems doing my usual activities
  - I have severe problems doing my usual activities

I am unable to do my usual activities

PAIN / DISCOMFORT

I have no pain or discomfort

I have slight pain or discomfort

I have moderate pain or discomfort

I have severe pain or discomfort

I have extreme pain or discomfort

ANXIETY / DEPRESSION

I am not anxious or depressed

I am slightly anxious or depressed

I am moderately anxious or depressed

I am severely anxious or depressed

I am extremely anxious or depressed

ADL: Please tick the ONE box that best describes your health before pandemic and TODAY.

Rank J1: Daily life is almost independent, and patients can go outside using different means of transportation without assistance from other individuals

Rank J2: Patients can go outside in the home vicinity without assistance from other individuals

Rank A1: Patients live independently indoors but require assistance to go out; moreover, they stay out of bed for most of the day

Rank A2: Patients live independently indoors but require assistance to go out; however, they seldom go out and take several bed rests during the day

Rank B: Patients require some assistance living indoors and spend most of the day in bed; however, they keep sitting up
